# Exploring the Cognitive Phenotypes of Implementation Barriers to Analgesia and Sedation Management Among ICU Nurses: A Q Methodology Study

**DOI:** 10.1155/jonm/3155537

**Published:** 2026-10-04

**Authors:** Huiqin Shi, Jingying Huang, Kailai Gu, Yunyi Zhong, Yilin Xu, Kaili Wang

**Affiliations:** ^1^ Intensive Care Unit Sir Run Shaw Hospital Zhejiang University School of Medicine Hangzhou China zju.edu.cn; ^2^ Zhejiang Key Laboratory of Precise Diagnosis and Treatment of Abdominal Infection Sir Run Shaw Hospital School of Medicine Zhejiang University Hangzhou Zhejiang 310016 China zju.edu.cn; ^3^ Nursing College Zhejiang Chinese Medical University Hangzhou China zcmu.edu.cn; ^4^ Operating Room Sir Run Shaw Hospital Zhejiang University School of Medicine Hangzhou China zju.edu.cn

**Keywords:** analgesia, factor analysis, intensive care unit, nurses, Q methodology, sedation

## Abstract

**Background:**

Despite the importance of “analgesia‐first” guidelines in intensive care units (ICUs), clinical implementation remains suboptimal due to systemic pressures and subjective dilemmas. However, it remains unclear how ICU nurses comparatively prioritize competing implementation barriers and whether shared patterns of viewpoints exist.

**Objective:**

This study aimed to explore shared patterns of ICU nurses’ subjective viewpoints regarding barriers to analgesia and sedation management using Q methodology.

**Methods:**

A Q methodology study was conducted from April 7 to May 7, 2026. The Consolidated Framework for Implementation Research (CFIR) informed development of the 50‐statement Q‐set and was subsequently used as an interpretive framework after factor extraction. Thirty purposively sampled ICU nurses completed the Q‐sort and brief postsort interviews. By‐person factor analysis was used to identify shared viewpoint patterns.

**Results:**

Three factors were identified, explaining 52.46% of the cumulative variance. Factor 1, “Professionally Confident and Responsibility‐Driven,” emphasized professional responsibility, confidence in analgesia and sedation management, and standardized, guideline‐informed practice. Factor 2, “Experience‐Driven with Perceived Organizational Support Gaps,” emphasized experiential clinical judgment alongside perceived limitations in previous formal preparation, departmental prioritization, and physician–nurse consensus. Factor 3, “Systemically Isolated and Communication‐Restricted,” highlighted communication‐related and contextual barriers, including perceived discrepancies between nurse and physician observations, handover discontinuities, environmental constraints, and patient/family‐related influences. As Factor 3 was defined by only two participants, this viewpoint should be considered preliminary. CFIR‐informed interpretation indicated that the three viewpoints reflected different configurations of implementation influences at the individual, organizational, interprofessional, and contextual levels.

**Conclusion:**

The identified heterogeneity in viewpoints suggests that uniform implementation approaches may not fully address the different barriers perceived by ICU nurses. Nursing leaders could consider strategies that are responsive to the implementation influences emphasized across different viewpoints, while the preliminary Factor 3 findings require confirmation in larger and more diverse samples.

## 1. Introduction

In the field of critical care medicine, standardized analgesia and sedation constitute a core pillar for ensuring patient safety and quality of care [1]. Intensive care unit (ICU) patients, subjected to the multiple aggressions of primary diseases, invasive procedures such as endotracheal intubation, and environmental deprivation, frequently suffer from severe pain and extreme anxiety [2]. Inadequate analgesia and sedation have been associated with hemodynamic instability and increased oxygen consumption [3, 4]. They may also increase the incidence of delirium [5] and contribute to postintensive care syndrome (PICS), including posttraumatic stress disorder (PTSD) [6, 7]. To ensure treatment safety and alleviate patient suffering, scientific analgesic and sedative therapy is particularly crucial [1, 2]. With the continuous evolution of analgesia and sedation concepts, the 2025 PADIS (Pain, Agitation/Sedation, Delirium, Immobility, and Sleep Disruption) guidelines emphasize an “analgesia‐first, goal‐directed sedation” approach [8]. This principle is also reflected in the early Comfort using Analgesia, minimal Sedatives and maximal Humane care (eCASH) concept [2], which aims to reduce nociceptive burden through appropriate analgesia while minimizing unnecessary sedation [1, 2, 8].

However, despite the widespread dissemination of standardized guidelines, their implementation in clinical ICU settings often remains suboptimal. Undersedation may be associated with adverse events such as unplanned extubation [5], whereas oversedation may contribute to prolonged mechanical ventilation and delayed early rehabilitation [9, 10]. This gap between evidence and practice is constrained by multidimensional and complex factors. Previous studies indicate that the chronic high workload and nursing staff shortages in the ICU severely compress the time window for nurses to perform high‐frequency, meticulous assessments [11, 12]. Meanwhile, within the context of China’s current health insurance policies, access to advanced objective monitoring technologies (such as the Bispectral Index, BIS) remains heavily contingent upon regional reimbursement coverage and the financial capacity of patients′ families. Furthermore, universal guidelines often reveal limitations when applied to highly heterogeneous subspecialty populations. For example, to avert cerebral metabolic crises, neurocritical care patients often struggle to routinely implement the “daily awakening” strategy recommended by the guidelines [10].

The aforementioned objective and complex barriers implicitly construct a highly stressful clinical practice environment. In this context, the implementation of management protocols is no longer the mechanical execution of medical orders but evolves into subjective trade‐offs and negotiations by healthcare professionals under multiple pressures. Research indicates that the correct implementation of analgesia and sedation management strategies by healthcare professionals is influenced by individual characteristics, the inner setting, the outer setting, and intervention characteristics [13]. The poor compliance with analgesia and sedation management is not merely a lack of knowledge but the multiple subjective dilemmas faced by healthcare professionals in complex, high‐pressure clinical scenarios [13]. These include the fear of adverse events such as accidental extubation, increased nursing workload due to cumbersome assessment tools, and the unclear allocation of decision‐making authority between physicians and nurses. Conversely, facilitators encompass robust collaboration within interdisciplinary teams, the widespread adoption of specialized training, and the enhancement of nurses’ autonomy in sedation titration [14, 15]. As the direct executors of analgesia and sedation protocols and the 24‐h dynamic monitors of patients’ vital signs, nurses play a central role in titrated medication administration, goal achievement, and multidisciplinary communication [16]. Clarifying nurses’ priority judgments and subjective trade‐offs regarding various barriers and facilitators may help inform improvement strategies.

Previous research examining barriers to analgesia and sedation management has primarily used cross‐sectional surveys and qualitative interviews [17–21]. These approaches have provided important insights into nurses’ knowledge, attitudes, practices, and experiences; however, they address different aspects of the implementation problem. Survey‐based approaches are generally structured around predefined variables, whereas qualitative interviews provide rich contextual accounts of individual experiences. Neither approach is specifically designed to examine how nurses prioritize competing barriers and facilitators relative to one another or to identify shared configurations of these subjective priorities across participants. Consequently, it remains unclear whether distinct patterns of viewpoints exist among ICU nurses and which implementation barriers are particularly salient within each pattern.

Q methodology is well suited to addressing this gap because it combines the systematic ranking of statements with by‐person factor analysis to identify shared patterns of subjective viewpoints [22, 23]. Building on our previous CFIR‐informed synthesis of multilevel barriers and facilitators to analgesia and sedation management [13], the present study used these identified barriers and facilitators to construct the Q‐set and examined how ICU nurses comparatively prioritized them. This approach enables the identification and interpretation of distinct shared viewpoint configurations and their most salient barriers and facilitators, thereby providing a more differentiated basis for considering implementation strategies.

## 2. Methods

### 2.1. Study Design

This study employed Q methodology following established methodological guidance [24]. Q methodology combines the systematic ranking of statements with by‐person factor analysis to identify shared patterns of subjective viewpoints across participants [22, 24]. It is particularly useful for examining how individuals comparatively prioritize and configure different perspectives on a given topic. Given the competing clinical and contextual considerations involved in analgesia and sedation management, Q methodology was used to explore shared patterns in ICU nurses’ perceptions of implementation barriers and facilitators. The study protocol was approved by the Ethics Committee of Sir Run Shaw Hospital, Zhejiang University School of Medicine (Approval No. 20260337).

### 2.2. Development of the Q‐Set

The CFIR framework provided the conceptual structure for developing the Q‐set. Barriers and facilitators identified in our previous CFIR‐informed mixed‐methods systematic review were organized according to relevant CFIR domains and constructs and then translated into an initial pool of 39 neutral, first‐person statements suitable for Q‐sorting. During refinement of the Q‐set, CFIR was also used to examine conceptual coverage so that the final statement set represented implementation influences arising from different levels of the clinical context rather than concentrating disproportionately on a single domain.

The preliminary 39‐item Q‐set was reviewed by a multidisciplinary panel of four ICU experts, including an experienced ICU clinical nurse, an ICU nurse educator, an ICU head nurse, and a senior ICU physician at the deputy chief physician level. Experts were selected based on having a master’s degree or higher, an intermediate or higher professional title, at least 5 years of ICU clinical experience, and relevant expertise in analgesia and sedation management, critical care nursing, or nursing management. Based on expert review, five double‐barrelled statements were split into separate single‐focus statements, resulting in a net increase of five statements. Six additional statements were also added to improve conceptual coverage, particularly within the CFIR domains of Intervention Characteristics, Inner Setting, and Characteristics of Individuals. These modifications resulted in a 50‐item Q‐set for pilot testing.

The 50‐item Q‐set was subsequently pilot‐tested with four ICU nurses to assess statement clarity, comprehensibility, and the feasibility of the Q‐sorting procedure. The pilot participants were able to complete the sorting procedure as intended but provided feedback on the wording of several statements and on the clarity of the sorting instructions. Based on this feedback, minor wording revisions were made to improve statement clarity, and the procedural instructions were further clarified to facilitate completion of the Q‐sort. No statements were added or removed, and the underlying conceptual content and CFIR mapping remained unchanged. The complete Q‐set is provided in Supporting Table S1, and its mapping to the CFIR domains and subconstructs is presented in Supporting Table S2.

### 2.3. Selection of the P Set

Purposive sampling was used to recruit ICU nurses with varied clinical ladder levels (N1–N4), professional titles, educational backgrounds, and years of ICU experience. Between April 7 and May 7, 2026, eligible participants were recruited from a tertiary Grade A hospital in Hangzhou according to the predefined inclusion and exclusion criteria. Recruitment information was distributed to potentially eligible ICU nurses through the department and nurse managers. Nurses who were interested in participating contacted the research team voluntarily; nurse managers were not involved in selecting or enrolling individual participants. Before participation, the study purpose and procedures were explained, and participants were informed that participation was voluntary and that they could decline or withdraw without any consequences. Written informed consent was obtained before data collection.

#### 2.3.1. Inclusion and Exclusion Criteria

1. **Inclusion criteria:** ① Possession of a registered nurse license and practicing certificate; ② ≥ 1 year of clinical nursing experience in the ICU; ③ an associate degree or higher in nursing; and ④ voluntary participation in this study.
(2) **Exclusion criteria:** ① Nursing staff on leave during the investigation period; ② visiting, probationary, or intern nurses; and ③ nursing administrators who were not routinely involved in ICU clinical practice, bedside clinical supervision, or clinical education related to patient care.

#### 2.3.2. Sample Size Estimation

In Q methodology, the P‐set is purposively selected to capture a range of viewpoints relevant to the research question rather than to achieve population representativeness or qualitative data saturation. Based on the commonly used methodological recommendation that the P‐set may comprise approximately one‐third to one‐half of the number of Q‐statements [24], the 50‐items Q‐set provided an initial reference range of 17–25 participants. We recruited 30 ICU nurses to include participants with varied clinical characteristics and to allow for potentially confounded or nondefining Q‐sorts.

### 2.4. Q‐Sorting Procedure

This study used a 9‐point (−4 to +4) quasinormal distribution grid to force‐sort the 50 Q‐statements. Before Q‐sorting, all participants received the same standardized instructions regarding the purpose, sorting steps, and use of the forced‐distribution grid to ensure procedural consistency (Supporting S3). Participants first preliminarily categorized the statement cards into three groups based on their subjective judgment: “generally agree”, “neutral”, and “generally disagree”. Subsequently, under the constraints of the grid capacity, they completed the final placement and sorting through repeated deliberation. Immediately following the Q‐sort, a brief postsort interview was conducted to support interpretation of each participant’s sorting decisions. The interview focused primarily on the reasons underlying the participant’s extreme placements (+4 and −4), with additional probes regarding difficult trade‐offs, relevant clinical experiences, and other considerations that influenced the Q‐sort (see Supporting Table S4 for the interview guide). As these interviews were intended to contextualize the Q‐sort rather than constitute standalone in‐depth qualitative interviews, they lasted approximately 5–10 min.

### 2.5. Statistical Analysis

Data analysis comprised descriptive statistics and Q‐sort analysis. Descriptive statistics were performed using SPSS software. Frequencies and percentages were calculated for participants’ sociodemographic and clinical characteristics. For the Q‐sort analysis, valid Q‐sorts were imported into R version 4.5.1, and a Pearson correlation matrix was constructed across participants. ① Principal component analysis (PCA) was used for initial factor extraction, with factors having eigenvalues > 1.0 considered for retention. ② Varimax orthogonal rotation was subsequently applied to obtain a more interpretable factor solution. A factor loading was considered statistically significant at *p* < 0.01 using the criterion of $\geq 2.58/\sqrt{N}$, where N represents the number of Q‐statements. Participants meeting the loading criterion for a factor were identified as defining sorts. Q‐sorts with significant loadings on more than one factor were considered confounded and were excluded from the subsequent weighted calculation of factor scores.

For each retained factor, defining Q‐sorts were weighted according to their factor loadings, and weighted statement scores were standardized as Z‐scores. These Z‐scores were subsequently converted into an idealized Q‐sort array representing the shared viewpoint associated with each factor. Factor interpretation and naming followed the statistical extraction and were not predetermined by the CFIR framework. For each factor, the overall factor array was examined with particular attention to distinguishing statements, extreme‐ranked statements, and cross‐factor consensus statements. Participants’ postsort explanations were used to contextualize the reasoning underlying salient statement placements. The salient statements within each factor were then mapped back to the relevant CFIR domains and constructs to identify the implementation influences emphasized within each viewpoint. CFIR was therefore used as an interpretive framework rather than as an a priori classification system for factor extraction.

Factor labels and characterizations were derived primarily from the configuration and relative salience of statements within each factor, together with participants’ postsort explanations. CFIR provided a conceptual structure for interpreting these configurations and describing how different implementation influences were combined within each shared viewpoint; the factor labels were not assigned directly from the CFIR domain or construct names. A CFIR‐informed characterization of the three factor viewpoints is provided in Supporting Table S5.

## 3. Results

A total of 30 participants were included in this study, and their sociodemographic characteristics are detailed in Table 1. The average time for respondents to complete the Q‐sorting procedure was approximately 24.6 min, and the postsort interviews averaged approximately 6.3 min. By‐person factor analysis identified three factors from the 30 Q‐sorts (Table 2). The cumulative variance explained by these three factors reached 52.46% (Factor 1: 31.03%; Factor 2: 14.47%; Factor 3: 6.96%), which is well above the empirical threshold of 35%∼40% recommended in Q‐methodology [24]. Furthermore, the composite reliabilities of the three factors were 0.99, 0.97, and 0.89, respectively, indicating high reliability of the factor arrays. Participant factor loadings and the statistical characteristics of the retained factors are presented in Tables 1 and 2, respectively.

**TABLE 1:** Demographic characteristics and factor loadings of participants.

ID	Gender	Education	Position	Experience		Nurse level	Factor 1	Factor 2	Factor 3
				ICU	Sedation & analgesia				
US1	Female	BSc	CN	12	12	N4	0.1798	0.6825^∗^	0.1202
US2	Male	BSc	CN	13	13	N4	0.7161^∗^	0.1797	−0.1887
US3	Female	MSc	HN	20	20	N4	0.5504	0.5849^∗^	0.0093
US4	Female	BSc	NE	12	12	N4	0.5391^∗^	0.4257	0.1188
US5	Female	BSc	CN	16	16	N4	0.7573^∗^	0.2478	0.4128
US6	Female	BSc	CN	4	4	N3	0.7573^∗^	0.2478	0.4128
US7	Female	MSc	CN	3	4	N2	0.2502	0.6643^∗^	−0.1243
US8	Female	MSc	CN	1	1	N1	0.5394^∗^	0.017	0.0231
US9	Male	MSc	CN	1	1	N1	0.0791	0.1228	−0.3748^∗^
US10	Female	BSc	CN	14	14	N4	0.2351	0.5939^∗^	−0.3677
US11	Female	BSc	CN	2	2	N3	−0.1213	0.6483^∗^	0.3068
US12	Female	MSc	CN	3	3	N3	0.5380^∗^	0.4069	0.2713
US13	Female	BSc	CN	9	9	N4	0.5493^∗^	0.2313	0.3926
US14	Male	BSc	CN	5	5	N4	−0.2712	0.4938^∗^	−0.1782
US15	Female	MSc	NE	12	12	N4	0.6586^∗^	0.2093	−0.2341
US16	Female	BSc	CN	6	6	N4	0.4813	0.5447^∗^	0.2246
US17	Female	BSc	CN	5	5	N3	0.3194	0.6674^∗^	−0.0141
US18	Male	BSc	CN	3	3	N3	0.3255^∗^	0.2075	−0.1076
US19	Female	BSc	CN	1	1	N1	0.6663^∗^	0.1416	−0.2314
US20	Female	BSc	CN	1	1	N1	0.6420^∗^	0.0578	0.0659
US21	Female	BSc	CN	11	11	N4	0.6733^∗^	0.3153	0.0394
US22	Female	BSc	HN	17	17	N4	0.7324^∗^	−0.0488	−0.0067
US23	Male	BSc	CN	4	4	N3	0.6785^∗^	0.2643	−0.3631
US24	Female	BSc	CN	15	15	N4	0.6470^∗^	0.2124	0.1964
US25	Female	BSc	CN	11	11	N4	0.7530^∗^	0.1959	0.1248
US26	Female	BSc	CN	9	9	N4	0.7981^∗^	−0.1138	−0.1038
US27	Female	BSc	CN	4	4	N2	0.0473	0.1907	0.7548^∗^
US28	Female	BSc	CN	1	1	N2	0.6125^∗^	0.3914	−0.1651
US29	Female	BSc	HN	16	16	N4	0.6918^∗^	0.2494	−0.2126
US30	Female	BSc	CN	7	7	N4	0.5217^∗^	0.3648	0.1462

*Note:* N1–N4, clinical nursing ladder system.

Abbreviations: BSc, Bachelor of Science; CN, clinical nurse; HN, head nurse; ICU, intensive care unit; MSc, Master of Science; NE, nurse educator.

^∗^Indicates a defining sort with a significant factor loading on that specific factor.

**TABLE 2:** Statistical characteristics of the extracted factors.

Factor characteristics	Factor 1	Factor 2	Factor 3
Significantly loaded participants (*n* = 30)	20	8	2
Eigenvalue	9.31	4.34	2.09
Explained variance (%)	31.03%	14.47%	6.96%
Cumulative explained variance (%)	31.03%	45.50%	52.46%
Composite reliability	0.99	0.97	0.89

Table 3 presents the Z‐scores and corresponding idealized Q‐sort values for all 50 Q‐statements across the three factors. Figure 1 provides a visual comparison of the 10 statements showing the largest between‐factor differences in standardized scores. These statements were identified according to the maximum range of Z‐scores across the three extracted factors. Supporting Figure S6 presents the idealized Q‐sort array for Factor 1.

**TABLE 3:** Comparison of Q‐sort values and Z‐scores across factors.

Statement	Factor 1		Factor 2		Factor 3		Significance
	Z‐score	Q‐sort value	Z‐score	Q‐sort value	Z‐score	Q‐sort value	
S1	0.22	0	0.09	0	−0.82	−2	F3 only
S2	0.22	0	1.05	2	0.94	2	F1 only
S3	−1.41	−3	−1.11	−2	−1.53	−4	Consensus
S4	1.03	2	1.82	4	−0.82	−2	Distinguish all
S5	1.4	3	0.78	1	−0.12	0	Distinguish all
S6	−0.86	−2	1.14	2	0.12	1	Distinguish all
S7	0.31	1	−0.99	−2	−0.71	−1	F1 only
S8	−0.92	−2	−0.68	−1	−0.47	−1	Consensus
S9	0.25	1	0.31	1	−0.94	−2	F3 only
S10	1.9	4	1.56	3	1.18	3	
S11	2.02	4	1.82	4	0.71	1	F3 only
S12	−0.76	−1	0.82	1	−0.35	−1	F2 only
S13	−1.88	−4	−1.36	−3	−2	−4	
S14	−1.74	−3	−1.42	−3	−1.41	−3	Consensus
S15	−1.64	−3	−1.11	−2	−0.12	0	Distinguish all
S16	−1.55	−3	−0.51	−1	0	0	F1 only
S17	−0.41	−1	−1.56	−3	0.7	1	Distinguish all
S18	1.34	3	1.05	2	−1.53	−3	F3 only
S19	0.62	1	1.44	3	2.23	4	Distinguish all
S20	−0.96	−2	−0.38	−1	0	0	F1 only
S21	−0.39	−1	0.07	0	−0.82	−2	F2 only
S22	0.72	2	0.03	0	−0.35	−1	F1 only
S23	1.18	2	−0.36	0	0.7	1	F2 only
S24	−0.25	−1	0.26	1	−1.3	−3	Distinguish all
S25	−0.18	0	−1.56	−4	0	0	F2 only
S26	0.12	0	−0.88	−2	0.94	2	Distinguish all
S27	−0.15	0	−0.21	0	1.53	3	F3 only
S28	0.38	1	−1.24	−2	0	0	F2 only
S29	0.83	2	−0.56	−1	−0.23	0	F1 only
S30	−0.28	−1	−0.15	0	−1.29	−2	F3 only
S31	1.72	3	0.95	1	0.47	1	F1 only
S32	0.2	0	−0.04	0	−0.47	−1	Consensus
S33	0.7	1	−0.83	−1	−0.12	0	F1 only
S34	−0.83	−2	−0.21	0	1.18	2	Distinguish all
S35	0.23	0	0.18	0	0.82	2	Consensus
S36	−1.24	−2	0.63	1	0	0	F1 only
S37	0.58	1	1.17	3	−1.53	−3	Distinguish all
S38	−1.99	−4	−1.13	−2	0.94	2	Distinguish all
S39	−0.08	0	−1.68	−4	0.7	1	Distinguish all
S40	0.51	1	0.35	1	−1.29	−2	F3 only
S41	0.32	1	1.75	3	1.65	3	F1 only
S42	0.22	0	1.08	2	−0.35	−1	F2 only
S43	−1.23	−2	−0.58	−1	2	4	Distinguish all
S44	0.22	0	1.01	2	1.41	3	F1 only
S45	−0.2	−1	−0.38	−1	0.35	1	Consensus
S46	−0.75	−1	0	0	1.18	2	Distinguish all
S47	1.23	3	0.59	1	−0.23	0	Distinguish all
S48	0.87	2	−0.8	−1	0.24	1	F2 only
S49	−0.47	−1	−1.27	−3	−0.59	−1	
S50	0.84	2	1.03	2	−0.59	−1	F3 only

*Note:* F1–F3, Factor 1 to Factor 3 (representing the three extracted cognitive phenotypes); S, statement number from the Q‐set used in this study (e.g., S1 indicates Statement 1); Z‐score, standardized score representing the weighted average placement of a statement for a specific factor; Q‐Sort Value, the idealized array score assigned to a statement within a specific factor (based on the quasinormal distribution grid); Consensus, consensus statements that do not significantly distinguish between any of the extracted factors; Distinguish all distinguishing statements that significantly differentiate all three factors; F1/F2/F3 only, statements that significantly distinguish one specific factor from the others.

**FIGURE 1:**
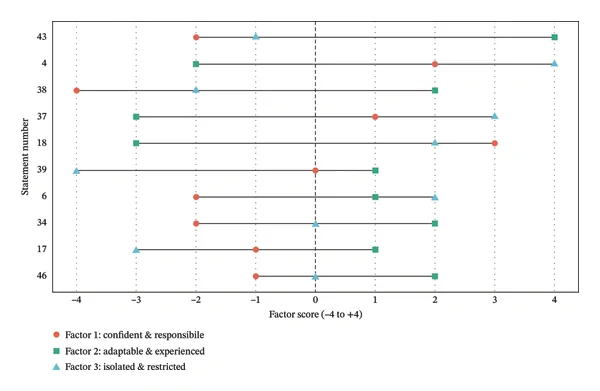
Comparison of the top 10 distinguishing Q‐statements across the three factors. Note: Factor 1 corresponds to the professionally confident and responsibility‐driven viewpoint; Factor 2 corresponds to the experience‐driven with perceived organizational support gaps viewpoint; and Factor 3 corresponds to the systemically isolated and communication‐restricted viewpoint.

### 3.1. Factor 1: Professionally Confident and Responsibility‐Driven

Factor 1 was defined by 20 participants and was characterized by strong professional responsibility and confidence. In their subjective sorting, they strongly agreed that analgesia and sedation management is a core nursing responsibility of profound significance (“Helping patients maintain effective analgesia and sedation is one of my job responsibilities,” +4; “It is very important to help patients achieve effective analgesia and sedation,” +4). They also expressed robust confidence in their professional assessment capabilities (“I am proficient in using sedation and analgesia assessment tools,” +3).

As Participant US26 emphasized during the postsort interview: *“Guidelines are constantly being updated. We cannot treat different patients based on subjective assumptions; we must rely on standardized protocols. Managing different patients based solely on personal subjective experience leads to nonstandardized care and hidden safety risks.”*

Notably, this group of nurses disagreed with compromising with objective environmental constraints. They strongly opposed attributing poor compliance to workload, expressing strong disagreement with objective barriers such as “Because I manage too many patients, I often cannot perform assessments at the required frequency” (−4), “Heavy basic nursing workloads make it difficult for me to find the time” (−3), or “Cumbersome paperwork takes up my time” (−3).

Furthermore, they clearly recognized the clinical benefits of analgesia and sedation. Participant US2 noted: *“Sedation and analgesia span the entire treatment process. Suboptimal outcomes will prolong the length of hospital stay and lead to prolonged bed occupancy, which inevitably exerts a negative impact on departmental performance.”* This indicates a high degree of intrinsic, responsibility‐driven motivation among these nurses.

Within the CFIR framework, Factor 1 was characterized primarily by individual‐level influences, particularly Knowledge and Beliefs, as reflected in participants’ strong endorsement of professional responsibility and competence in analgesia and sedation management (S5, S10, and S11). In contrast, several structural constraints within the Inner Setting, including workload‐ and time‐related barriers, were assigned relatively low importance within this viewpoint. Together with the broader factor configuration and postsort explanations, these patterns informed the characterization of Factor 1 as “Professionally Confident and Responsibility‐Driven.”

### 3.2. Factor 2: Experience‐Driven With Perceived Organizational Support Gaps

Factor 2 comprised eight defining participants and was characterized by a strong reliance on experiential clinical judgment alongside perceived gaps in formal preparation and organizational support for analgesia and sedation management. Participants strongly endorsed clinical experience as an important basis for decision‐making (*“I believe rich clinical experience is the most important foundation for making good analgesia and sedation decisions,”* S4, +4). They also endorsed situational adjustment when managing unstable patients (*“When vital signs are extremely unstable, I will temporarily deprioritize sedation and analgesia adjustments,”* S41, +3), reflecting a tendency to adapt management priorities according to the immediate clinical condition.

At the same time, participants perceived limitations in their formal preparation. They strongly disagreed that previous education and workplace training had fully prepared them for analgesia and sedation management (S39, −4), while positively endorsing regular knowledge and skills assessments as a means of improving their management capabilities (S37, +3). Physical exhaustion was also perceived as affecting their willingness to engage in further learning after work (S6, +2). As Participant US1 explained: “*As a senior nurse, the degree of work fatigue directly determines the willingness to learn. When physically exhausted, I must sleep after work and cannot stay up late reading new literature as I used to.*”

Perceived support gaps were also evident at the organizational and interprofessional levels. Participants strongly disagreed that high‐quality analgesia and sedation management was regarded as an absolute departmental priority (S25, −4) and disagreed that clear consensus with physicians regarding sedation goals could always be achieved (S17, −3). One participant emphasized the complementary roles of nurses and physicians in addressing these challenges: “*Nurses can compensate for doctors′ blind spots by capturing patients’ pain behaviors through prolonged observation; meanwhile, doctors can provide superior pharmacological knowledge… Mutual complementation between both parties is best for the patient.*” Taken together, this factor reflects an experience‐driven approach to clinical decision‐making accompanied by perceived limitations in formal preparation, departmental prioritization, and interprofessional support.

Within the CFIR framework, Factor 2 reflected influences across the Characteristics of Individuals, Inner Setting, and Process domains. Reliance on clinical experience and the influence of fatigue on learning were mapped to Other Personal Attributes (S4 and S6). Perceived difficulty reaching physician–nurse consensus was mapped to Networks and Communications (S17), low perceived departmental prioritization to Implementation Climate (S25), and perceived insufficiency of previous preparation to Readiness for Implementation (S39). The positive valuation of regular competency assessment and situational adjustment of analgesia and sedation management were further represented within the Process domain through Reflecting and Evaluating (S37) and Executing (S41), respectively. This configuration informed the characterization of Factor 2 as “Experience‐Driven with Perceived Organizational Support Gaps”.

### 3.3. Factor 3: Systemically Isolated and Communication‐Restricted

Factor 3 was defined by two participants whose Q‐sorts showed a similar pattern characterized by perceived barriers in interprofessional communication and the surrounding clinical environment. Given the small number of defining participants, this factor is interpreted cautiously as a preliminary shared viewpoint rather than as a representative profile of a broader subgroup.

Both participants strongly endorsed perceived discrepancies between nurses’ continuous bedside observations and physicians’ more intermittent assessments (S19, +4), suggesting that differences in observation patterns may contribute to feelings of being misunderstood during analgesia and sedation management. They also perceived several external constraints, including interference related to limited understanding among patients or family members (S43, +4) and limitations in the ICU physical environment for close observation of sedation status (S27, +3).

Communication‐related concerns were also reflected in their disagreement that analgesia and sedation information was always comprehensively transmitted during shift handovers (S24, −3). In addition, they did not strongly endorse some conventional implementation approaches, including joint physician–nurse ward rounds (S18, −3) and regular knowledge and skills assessments (S37, −3). Taken together, these two Q‐sorts suggest a viewpoint in which communication difficulties and perceived contextual constraints are prominent; however, this pattern requires cautious interpretation because of the limited number of defining participants.

Within the CFIR framework, Factor 3 reflected communication‐ and context‐related influences spanning the Inner Setting, Outer Setting, and Process domains. Concerns regarding physician–nurse communication, joint rounds, and continuity of information during handovers were mapped to Networks and Communications (S18, S19, and S24), while constraints related to ICU layout were mapped to Structural Characteristics and Resources (S27). The perceived influence of patients’ or family members’ limited understanding of the clinical situation was mapped to Patient Needs and Resources within the Outer Setting (S43). The low perceived usefulness of regular competency assessment was represented within the Process domain through Reflecting and Evaluating (S37). Together, these patterns informed the characterization of Factor 3 as “Systemically Isolated and Communication‐Restricted.” Given that this factor was defined by only two participants, this characterization should be interpreted as preliminary.

### 3.4. Cross‐Factor Consensus

Despite the differences among the three factor viewpoints, several Q‐statements did not significantly distinguish between factors. As shown in Table 3, S3, S8, S14, S32, S35, and S45 were identified as consensus statements. Consensus in this context indicates the absence of statistically significant between‐factor differences rather than identical levels of agreement or disagreement across all three factor arrays. Among these statements, all three factors disagreed that the current clinical assignment model made it difficult to guarantee sufficient time for standardized assessment (S14; Factor 1 = −3, Factor 2 = −3, Factor 3 = −3). They also disagreed with the statement that they lacked confidence when managing analgesia and sedation in patients with unstable vital signs (S3; Factor 1 = −3, Factor 2 = −2, Factor 3 = −4).

In addition, although S13 was not identified as a formal consensus statement in Table 3, all three factor arrays placed it on the disagreement side, suggesting that excessive patient load was not perceived as a prominent barrier to performing assessments at the required frequency within this study sample. These shared patterns should be interpreted as features of the present single‐center sample rather than as evidence that workload or staffing constraints are unimportant in other ICU settings. Table 4 provides a comparative summary of the characteristics, salient implementation concerns, and potential implementation considerations associated with the three viewpoints.

**TABLE 4:** Summary of factor characteristics, salient implementation concerns, and potential implementation considerations across the three viewpoints.

Factor viewpoint	Confident and responsible	Adaptable and experienced	Isolated and restricted
Descriptive visual profile	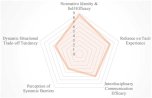	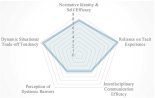	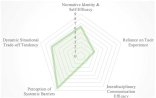

Defining participants	20 defining participants	8 defining participants	2 defining participants; interpretation should be considered preliminary

Characteristics	• Strong professional responsibility and confidence in analgesia and sedation management. • Strong endorsement of the importance of effective analgesia and sedation and perceived competence in using assessment tools. • Workload‐ and time‐related structural constraints were comparatively less salient within this viewpoint.	• Strong reliance on experiential clinical judgment and situational adaptation. • Perceived gaps in previous formal preparation and organizational support. • Despite perceived limitations in prior preparation, participants positively valued regular competency assessment.	• Communication‐ and context‐related barriers were particularly salient. • Emphasis was placed on discrepancies between nurses’ continuous bedside observations and physicians’ more intermittent assessments. • Environmental constraints, handover discontinuities, and patient/family‐related influences were also prominent.

Salient implementation concerns	• Managing analgesia and sedation primarily on the basis of subjective assumptions was viewed negatively. • Excessive patient load and paperwork were not prioritized as major barriers to standardized assessment (S13, −4; S15, −3).	• Previous education and workplace training were perceived as insufficient preparation for practice (S39, −4). • High‐quality analgesia and sedation management was not perceived as an absolute departmental priority (S25, −4). • Clear physician–nurse consensus regarding sedation goals was perceived as difficult to achieve consistently (S17, −3). • Physical exhaustion was perceived as affecting willingness to engage in further learning after work (S6, +2).	• Perceived discrepancies between nurse and physician observations (S19, +4). • Influence of limited understanding among patients or family members (S43, +4). • ICU physical‐layout constraints (S27, +3). • Incomplete analgesia and sedation information during shift handovers (S24, −3). • Relatively low endorsement of joint physician–nurse ward rounds and regular competency assessment (S18, −3; S37, −3).

Salient priorities	• Maintaining safe and effective analgesia and sedation was regarded as an important nursing responsibility (S10, +4; S11, +4). • Standardized assessment and guideline‐informed practice were emphasized.	• Clinical experience was regarded as an important foundation for analgesia and sedation decision‐making (S4, +4). • Situational adjustment of management priorities during marked physiological instability was endorsed (S41, +3). • Continued knowledge and skills assessment was positively valued (S37, +3).	• Greater emphasis was placed on communication continuity and contextual barriers than on some conventional individual‐focused implementation approaches. • Timely and accurate information sharing appeared particularly relevant within this viewpoint.

Potential implementation considerations	• Clinical empowerment and opportunities to participate in quality‐improvement activities could be considered. • Peer‐support and clinical education roles may provide opportunities to draw on the professional responsibility and confidence represented within this viewpoint.	• Practice‐oriented professional development could build on experiential clinical knowledge while supporting its alignment with current evidence. • Scenario‐based learning, reflective case discussions, and peer exchange may provide useful approaches for supporting continued learning and shared clinical reasoning.	• Strategies aimed at strengthening structured physician–nurse communication may warrant further investigation. • Improving continuity of analgesia and sedation information during handovers and facilitating timely access to shared clinical information may also be relevant. • Given that this factor was defined by only two participants, these implications should be considered exploratory rather than prescriptive.

*Note:* S, statement number from the Q‐set used in this study. The radar charts provide a descriptive visual summary of five interpretive dimensions derived from representative Q‐statements. These dimensions were developed for visualization purposes and do not represent formal CFIR constructs or additional psychometric scales. Scores were derived from the factor‐level Q‐sort values reported in Table 3. For dimensions comprising multiple statements, the corresponding Q‐sort values were averaged. Statement S19 was reverse‐coded for Interdisciplinary Communication Efficacy, and S25 was directionally reverse‐coded for Dynamic Situational Trade‐off Tendency so that higher scores consistently represented a greater level of the respective dimension. A constant of 5 was then added to each resulting value to facilitate visualization on a positive scale. The five dimensions were based on the following statements: Normative Identity and Self‐Efficacy (S5, S10, S11); Reliance on Tacit Experience (S4); Interdisciplinary Communication Efficacy (S18, reverse‐coded S19); Perception of Systemic Barriers (S27, S43); and Dynamic Situational Trade‐off Tendency (S41, directionally reverse‐coded S25). These descriptive scores were used only for visualization and were not used for factor extraction, factor naming, or CFIR‐based interpretation. Factor 3 was defined by only two participants; therefore, its characterization and associated implementation considerations should be regarded as preliminary.

Abbreviation: ICU, intensive care unit.

## 4. Discussion

Through Q methodology, this study identified three shared patterns of subjective viewpoints regarding barriers to analgesia and sedation management among ICU nurses, highlighting heterogeneity in how implementation challenges were perceived and prioritized. Although standardized assessment tools (e.g., the Richmond Agitation–Sedation Scale [RASS] and Critical‐Care Pain Observation Tool [CPOT]) and bundled approaches such as the Assess, prevent, and manage pain; Both spontaneous awakening trials and spontaneous breathing trials; Choice of analgesia and sedation; Delirium assess, prevent, and manage; Early mobility and exercise; Family engagement and empowerment (ABCDEF) bundles have been widely promoted in critical care practice, their implementation may be shaped by the complex and dynamic conditions of the ICU [17, 25, 26]. Previous research has similarly suggested that the translation of standardized guidance into clinical practice is influenced by practitioners’ interpretations of local clinical conditions and implementation contexts [27]. In the present study, these differences were reflected in the distinct configurations of Q‐statements that characterized the three extracted viewpoints. When interpreted through the CFIR after factor extraction, the three viewpoints reflected different configurations of implementation influences rather than mapping onto single CFIR domains. Factor 1 was characterized predominantly by individual‐level influences, whereas Factors 2 and 3 reflected additional influences related to the Inner Setting and implementation Process, with Outer Setting influences also evident in Factor 3.

Comparison of the factor arrays showed marked differences across the three viewpoints on several clinically relevant statements. Among these, S37, S43, and S4 showed particularly pronounced between‐factor differences and helped differentiate the implementation concerns represented by the three viewpoints. First, regarding attitudes toward “competency assessment,” participants defining Factor 2 strongly endorsed the value of regular assessments, viewing them as an effective avenue to enhance practical skills. Compared with Factor 2, the two participants defining Factor 3 placed less value on regular knowledge and skills assessments and greater emphasis on communication‐related and contextual barriers. They also regarded interference related to limited understanding among patients or family members as particularly salient. This contrast suggests that implementation barriers may be perceived differently across viewpoints, with communication processes and external contextual conditions being more prominent within the perspective represented by Factor 3 [26]. Although both defining participants were relatively junior nurses, this demographic pattern should be interpreted cautiously and should not be taken to indicate that these concerns are characteristic of junior or novice ICU nurses more broadly. Rather, the findings suggest a preliminary viewpoint that warrants further examination in larger and more diverse samples. These findings suggest that departments could consider strengthening access to senior clinical support for nurses encountering complex patient–family or interprofessional communication challenges. As suggested by Li et al. [28], such support could include case‐based guidance, communication assistance, and timely escalation of complex concerns. Drawing on the clinical experience of senior staff may help nurses navigate these challenging situations and support the implementation of analgesia and sedation practices [29].

Participants defining Factor 1 emphasized professional responsibility, confidence, patient safety, standardized assessment, and guideline‐informed practice. Their relatively low ranking of workload‐ and time‐related constraints suggests that these barriers were less salient within this viewpoint rather than absent in practice. Implementation approaches for this viewpoint could consider clinical empowerment, participation in quality‐improvement activities, and opportunities for peer‐support or clinical education [30–32]. Providing access to updated academic resources and opportunities to participate in departmental quality‐improvement activities may further support their involvement in the local adaptation of clinical guidance. Incorporating frontline implementation experience into such activities could also help improve the contextual relevance of analgesia and sedation practices.

Participants defining Factor 2 (“Experience‐Driven with Perceived Organizational Support Gaps”) demonstrated a strong reliance on experiential clinical judgment. Such experiential and heuristic decision‐making can be highly valuable in critical care, particularly when rapid responses are required in complex and dynamic situations. Rather than viewing these experience‐based practices as barriers to be overcome, they should be regarded as important forms of tacit clinical knowledge. The implementation challenge lies not in replacing experiential judgment but in supporting its integration with current evidence‐based recommendations and making valuable experiential knowledge more accessible within the clinical team. For example, reflective case discussions, scenario‐based learning, and peer exchange may provide opportunities for experienced nurses to articulate the reasoning underlying their clinical decisions while also engaging with updated guidelines and standardized care pathways. Such approaches may help preserve the strengths of experiential expertise while promoting shared learning and consistency in analgesia and sedation practice.

Importantly, participants defining Factor 2 did not reject training or competency assessment. They strongly disagreed that previous education and workplace training had fully prepared them for analgesia and sedation management (S39, −4), while positively endorsing regular knowledge and skills assessments as a means of improving their management capabilities (S37, +3). This pattern suggests a perceived need for continued, practice‐oriented professional development rather than resistance to training itself. This interpretation is broadly consistent with previous qualitative work describing challenges and barriers to optimizing sedation practice in ICU settings [21]. Accordingly, implementation approaches for this viewpoint could consider building on nurses’ experiential clinical knowledge while supporting its alignment with current evidence and shared clinical practice. This is also consistent with the broader literature examining barriers and facilitators to clinical practice guideline implementation among nurses in emergency and critical care settings [32]. Scenario‐based learning and reflective debriefing may provide potential approaches for facilitating such practice‐oriented learning and reflection [33].

Within the CFIR Inner Setting domain, the two participants defining Factor 3 placed particular emphasis on communication‐related and contextual barriers. Their Q‐sorts suggested perceived discrepancies between nurses’ continuous bedside observations and physicians’ more intermittent assessments, together with concerns about incomplete handover information and external interference from patients or family members. These findings are consistent with the broader importance of interprofessional communication and information continuity in analgesia and sedation management [13, 34, 35]. However, Factor 3 was defined by only two participants. Although both were relatively junior nurses, this demographic characteristic should not be interpreted as indicating that the identified viewpoint is characteristic of junior or novice ICU nurses more broadly. Rather, the factor should be regarded as a preliminary perspective suggesting that, for some nurses, communication processes and contextual conditions may be particularly salient barriers to analgesia and sedation implementation.

From an implementation perspective, these findings suggest that strategies aimed at strengthening structured physician–nurse communication, improving continuity of analgesia and sedation information during handovers, and facilitating timely access to shared clinical information may warrant further investigation. Given the limited number of defining participants, these implications should be considered exploratory rather than prescriptive and require confirmation in larger and more diverse samples. Future research could further examine whether digital decision‐support approaches, including artificial intelligence (AI)‐assisted tools, may facilitate these communication and information‐sharing processes; however, such technologies were not evaluated in the present study.

Notably, the cross‐factor consensus identified in this study differed from some previous reports in which staffing shortages and workload were described as prominent barriers to analgesia and sedation management [18, 21]. Across all three factors, participants disagreed that excessive patient load (S13) or the current clinical assignment model (S14) prevented them from performing standardized assessments. Similar patterns were observed for paperwork‐related demands (S15), although the strength of disagreement varied across factors. These findings suggest that, within this study setting, workload‐related constraints were not among the most salient perceived barriers to analgesia and sedation implementation. However, this should not be interpreted as evidence that staffing or workload is unimportant in other ICU settings. Differences between the present findings and previous studies may reflect contextual variation in staffing models, workflow organization, documentation systems, or local implementation environments and therefore warrant further investigation [36, 37]. Participants across factors also expressed confidence in managing patients with unstable vital signs. Rather than indicating a general level of “clinical resilience,” this consensus may more cautiously be interpreted as reflecting shared perceived confidence in this aspect of analgesia and sedation‐related clinical decision‐making.

From a macroscopic implementation and evaluation perspective, incorporating postsort interview data, respondents also revealed a significant tension between theoretical frameworks and real‐world execution. On one hand, multiple respondents mentioned the potential value of establishing critical care subspecialty groups, but all pointed out that current subspecialty construction remains in its infancy due to a lack of top‐level institutional support, meaning theoretical advantages have not yet translated into systemic driving forces. On the other hand, similar to previous research findings, there is a high degree of heterogeneity in analgesia and sedation outcome indicators and an absence of standardized verification mechanisms [38]. A unified baseline for assessment is often lacking between different levels of hospitals and even among different critical care subspecialties within the same hospital [17]. This lack of cross‐specialty standardized quality control tools makes it difficult for hospital administrators to conduct horizontal benchmarking and effective oversight of implementation fidelity across the entire institution [35]. Future work could examine whether stronger organizational support for critical care subspecialty groups and the development of a feasible, context‐sensitive core indicator set may facilitate benchmarking, clinical audit, and feedback in analgesia and sedation management.

## 5. Limitations

This study has several limitations. First, Q methodology is designed to identify shared patterns of subjective viewpoints rather than to estimate their prevalence in a broader population. Therefore, the factors identified in this study should not be interpreted as representing fixed categories of ICU nurses or as indicating the proportional distribution of these viewpoints among ICU nurses more generally. Second, the findings are based on participants’ self‐reported subjective priorities rather than direct observation of clinical behavior. Q‐sorts and postsort interviews may therefore be influenced by self‐presentation or social desirability, and participants’ stated priorities may not necessarily correspond to their actual decisions in practice. In addition, the researcher‐facilitated postsort interviews and the subsequent interpretation and naming of factors involve a degree of researcher judgment and may be subject to interviewer or interpretive bias. The controlled Q‐sorting environment also differs from real‐world ICU decision‐making, where nurses work under time pressure, uncertainty, competing clinical demands, and rapidly changing patient conditions. Consequently, the identified factor configurations should be interpreted as shared subjective viewpoints rather than direct representations of actual bedside decision‐making behavior.

Third, Factor 3 was defined by only two participants. Although their Q‐sorts showed a sufficiently similar pattern to contribute to the extracted factor, the small number of defining sorts limits confidence in the stability and transferability of this viewpoint. In particular, the fact that both participants were relatively junior nurses should not be interpreted as indicating that this perspective is representative of junior ICU nurses more broadly. The interpretation and implementation implications associated with Factor 3 should therefore be considered preliminary and require confirmation in larger and more diverse samples. Finally, this was a single‐center study using purposive sampling, and the identified viewpoints may have been influenced by the specific organizational and clinical context of the participating ICU. Future multicenter studies with larger and more diverse samples would be valuable for examining the transferability of these findings. Future research could also include nurses from different critical care subspecialties, such as neurological and cardiovascular surgical ICUs, to examine whether similar viewpoint configurations are identified across different clinical contexts.

## 6. Conclusion

Through Q methodology, this study identified three shared patterns of subjective viewpoints regarding barriers to analgesia and sedation management among ICU nurses. These findings suggest that implementation approaches may benefit from considering heterogeneity in nurses’ perceived barriers and priorities. Rather than relying exclusively on uniform training approaches, nursing leaders could consider strategies responsive to the different implementation influences emphasized across the identified viewpoints.

**Nomenclature**
ABCDEF: Assess, prevent, and manage pain; Both spontaneous awakening trials and spontaneous breathing trials; Choice of analgesia and sedation; Delirium assess, prevent, and manage; Early mobility and exercise; Family engagement and empowerment
AI: Artificial intelligence
BIS: Bispectral Index
CFIR: Consolidated Framework for Implementation Research
CPOT: Critical‐Care Pain Observation Tool
eCASH: Early Comfort using Analgesia, minimal Sedatives and maximal Humane care
GAI: Generative artificial intelligence
ICU: Intensive care unit
PADIS: Pain, Agitation/Sedation, Delirium, Immobility, and Sleep Disruption
PCA: Principal component analysis
PICS: Postintensive care syndrome
PTSD: Posttraumatic stress disorder
RASS: Richmond Agitation–Sedation Scale
SPSS: Statistical Package for the Social Sciences

## Funding

This research was supported by grants from the Department of Zhejiang Health Science and Technology Project (Grant No. 2025KY081).

## Consent

Informed consent was obtained from all participants prior to their participation in the study.

## Conflicts of Interest

The authors declare no conflicts of interest.

## Supporting Information

Additional supporting information can be found online in the Supporting Information section.

## Supporting information


**Supporting Information** Supporting File 1. Additional materials for this Q methodology study, including the 50‐item Q‐set and statement orientations (Table S1), mapping of Q‐set statements to CFIR domains and subconstructs (Table S2), standardized participant instructions for the Q‐sorting procedure (Supporting S3), postsort semistructured interview guide (Table S4), CFIR‐informed characterization of the three factor viewpoints (Table S5), and the idealized Q‐sort array for Factor 1 (Supporting S6).

## Data Availability

The data that supports the findings of this study are available in the Supporting Information of this article.
