# Supplementary material for: Exploring the Cognitive Phenotypes of Implementation Barriers to Analgesia and Sedation Management Among ICU Nurses: A Q Methodology Study
Source: J Nurs Manag. 2026 Oct 4;2026:3155537. doi: 10.1155/jonm/3155537 (PMC13635794; doi:10.1155/jonm/3155537)
Supplement: Supplementary file 1 [file JONM-2026-3155537-s001.zip › Revised Supplementary .docx]

**Supplementary Table S1:The 50-Item Q-Sample Statements and Their Orientations.**

| **No.** | **Statement** | **Orientation** |
| --- | --- | --- |
| 1 | I am confident in my skills to assess patients' pain levels. | Positive |
| 2 | I am confident in my skills to assess patients' depth of sedation. | Positive |
| 3 | I sometimes lack confidence when managing analgesia and sedation for patients with unstable vital signs. | Negative |
| 4 | I believe rich clinical experience is the most important foundation for making good analgesia and sedation decisions. | Positive |
| 5 | I am proficient in using sedation and analgesia assessment tools (e.g., RASS, CPOT). | Positive |
| 6 | Physical exhaustion directly affects my willingness to learn new knowledge about analgesia and sedation after work. | Negative |
| 7 | I am satisfied with the sense of professional fulfillment brought by my nursing career. | Positive |
| 8 | I believe analgesia and sedation management is unrelated to departmental performance evaluation and income. | Negative |
| 9 | I have a clear grasp of the usage and side effects of commonly used analgesic and sedative drugs in our department. | Positive |
| 10 | Helping patients maintain effective analgesia and sedation is one of my job responsibilities. | Positive |
| 11 | It is very important to help patients achieve effective analgesia and sedation. | Positive |
| 12 | Prolonged physical exertion during work makes it difficult for me to maintain optimal assessment ability and focus on the end of my shift. | Negative |
| 13 | Because I manage too many patients, I often cannot perform assessments at the required frequency. | Negative |
| 14 | The department's current clinical assignment model makes it difficult to guarantee time for standardized assessments. | Negative |
| 15 | Cumbersome paperwork takes up my time for direct observation and patient care. | Negative |
| 16 | Heavy basic nursing workloads make it difficult for me to allocate sufficient time for standardized analgesia and sedation management. | Negative |
| 17 | I can always reach a clear consensus with doctors on sedation and analgesia goals. | Positive |
| 18 | Joint physician-nurse ward rounds are very helpful for developing analgesia and sedation plans. | Positive |
| 19 | We observe the patient's immediate agitation, whereas physicians observe the depth of sedation during ward rounds; this discrepancy often leads to us being misunderstood. | Negative |
| 20 | Nurses' professional opinions in analgesia and sedation management are not sufficiently respected. | Negative |
| 21 | When I have different views on a sedation plan, I can raise them with the physician without any pressure. | Positive |
| 22 | Nursing managers provide us with professional guidance to improve our analgesia and sedation management capabilities. | Positive |
| 23 | Department leaders explicitly emphasize the importance of standardized analgesia and sedation management. | Positive |
| 24 | During shift handovers, information regarding patients' analgesia and sedation is always comprehensively and accurately transmitted. | Positive |
| 25 | The department views high-quality analgesia and sedation management as an absolute work priority. | Positive |
| 26 | The noise level in the ICU frequently affects patients' ability to maintain an ideal state of sedation. | Negative |
| 27 | The layout and spatial design of the ICU are not conducive to my close observation of patients' sedation status. | Negative |
| 28 | The analgesia and sedation sub-specialty group established by the department provides important professional support for my clinical practice. | Positive |
| 29 | When I encounter management difficulties, I can usually get help from senior nurses or experts. | Positive |
| 30 | The equipment used, such as analgesia and sedation pumps, is always reliable in performance and smooth to operate. | Positive |
| 31 | I find that analgesia and sedation medical orders sometimes need to be adjusted based on the specific observed conditions of the patient. | Positive |
| 32 | Regularly published quality data, such as the departmental delirium incidence rate, prompts me to improve my practice. | Positive |
| 33 | I can access clear, executable, and easy-to-understand analgesia and sedation management protocols in the department. | Positive |
| 34 | Existing analgesia and sedation management protocols sometimes conflict with actual emergency life-saving interventions. | Negative |
| 35 | Requiring everyone to use the same assessment flow sheet is important and beneficial. | Positive |
| 36 | Some guideline recommendations are difficult to implement in our department's clinical environment. | Negative |
| 37 | Regular knowledge and skills assessments are an effective way to improve my personal analgesia and sedation management capabilities. | Positive |
| 38 | Regular analgesia and sedation-related training held by the hospital adds a lot of burden to my daily work. | Negative |
| 39 | The training I received in school and at work has fully prepared me to handle analgesia and sedation management. | Positive |
| 40 | I can accurately select the appropriate pain or sedation assessment tools based on the patient's current varying conditions. | Positive |
| 41 | When vital signs are extremely unstable, I will temporarily deprioritize sedation and analgesia adjustments. | Neutral |
| 42 | I am adept at judging whether a patient is uncomfortable by observing their body language and facial expressions. | Positive |
| 43 | The lack of understanding of dynamic disease treatment knowledge among patients or their families interferes with clinical nurses' selection of analgesia and sedation strategies. | Negative |
| 44 | Having cared for a large number of critically ill patients makes me more confident when facing analgesia and sedation challenges. | Positive |
| 45 | I believe the results obtained using pain scales truly reflect the patients' feelings. | Positive |
| 46 | I believe excessively frequent sedation assessments have limited actual benefits for some patients. | Negative |
| 47 | Seeing patients comfortable due to my management is one of the sources of satisfaction in my work. | Positive |
| 48 | I can receive specific feedback and guidance on my practice from senior colleagues or managers. | Positive |
| 49 | I worry that strictly implementing standardized protocols will limit my flexibility in handling individual differences. | Negative |
| 50 | Participating in specialized training effectively enhances my understanding and attitude toward analgesia and sedation management. | Positive |

**Supplementary Table S2. Mapping of the Q-Set Statements to the CFIR Domains and Sub-constructs**

| CFIR Domains | CFIR Sub-constructs | Q-set Statements |
| --- | --- | --- |
| Characteristics of Individuals | Knowledge & Beliefs | S5、S9、S10、S11、S45、S46 |
|  | Self-efficacy | S1、S2、S3、S40、S42、S44 |
|  | Other Personal Attributes | S4、S6、S7、S12、S47 |
| Inner Setting | Structural Characteristics & Resources | S13、S14、S15、S16、S26、S27、S30 |
|  | Networks & Communications | S17、S18、S19、S20、S21、S24 |
|  | Implementation Climate | S8、S25、S29、S34 |
|  | Readiness for Implementation | S22、S23、S38、S39、S50 |
| Intervention Characteristics | Adaptability | S31、S49 |
|  | Complexity | S36 |
|  | Design Quality & Packaging | S33、S35 |
| Outer Setting | Patient Needs & Resources | S43 |
| Process | Engaging | S28 |
|  | Executing | S41 |
|  | Reflecting & evaluating | S32、S37、S48 |

**Supplementary S3. Standardized Participant Instructions for the Q-Sorting Procedure.**

**Survey on ICU Nurses’ Perspectives on Sedation and Analgesia Management**

**— Q-Sorting Operational Guide**

Dear Colleagues：

Thank you for participating in this research! This study aims to understand the genuine perspectives	and	feelings	of	clinical	nurses regarding	“ICU	sedation	and	analgesia management” . Please note: This sorting activity is not an exam; there are no standard answers, nor is there a “right” or “wrong” . We are solely seeking your authentic personal views and work experiences.

**Preparation：**

You have a stack of cards in front of you (50 cards in total), and each card features a statement regarding factors that influence clinical nurses in the implementation of sedation and analgesia management.

There is a pyramid-shaped distribution grid paper (ranging from -4 to +4) containing a total of 50 blank spaces.

**Specific** **Operations：**

**Step** **1:** **Read** **Through** **and** **Initial** **Classification**

Please first briefly browse the Q-Sample summary in Table 1 on pages 3-4 to get an initial impression of the card contents.

Based on your first instinct, divide the cards into three piles:

- Right Pile (Agree): Cards you believe align with your views or truly reflect occurrences in your work.
- Left Pile (Disagree): Cards you believe do not align with your views or are not prominent in your work.
- Middle Pile (Neutral/Uncertain): Cards you feel are ambiguous, situational, or simply not important to you.

(Note: You do not need to count the exact number of cards in this step; simply divide them based on your intuition.)

**Step** **2:** **Detailed** **Sorting**

Now, please place these three piles of cards into the grid on the paper. You must fill every single space, and each space can only accommodate one card.

Suggested Operation Sequence: Sort from the two extremes towards the center.

- Pick the extremes first: From the “Agree” pile, pick out the 2 cards you agree with the most strongly and place them in the spaces on the far right (+4).
- From the “Disagree” pile, pick out the 2 cards you strongly disagree with (or dislike the most) and place them in the spaces on the far left (-4).
- Move inwards: Next, from the remaining “Agree” pile, select 4 cards you relatively agree

with and place them in the (+3) positions.

From the remaining “Disagree” pile, select 4 cards you relatively disagree with and place them in the (-3) positions.

Continue filling: Proceed to place the cards according to the required quantities: 6 cards for (+2), 6 cards for (-2), 8 cards for (+1), and 8 cards for (-1).

Process remaining cards: Finally, please place all the remaining 10 cards (usually the ones you feel are less important or are uncertain about) into the middle (0) spaces.

**Step** **3:** **Review** **and** **Adjustment**

Please take a look at the entire distribution map.

Adjustment: If you feel a card’s position is not quite right, you can move or swap its position at any time until the map perfectly represents your viewpoint.

(Note: There is not top-to-bottom ranking within the same column; as long as cards are in the same column, they carry the same weight.)

**Step** **4:** **Recording** **and** **Interview**

After confirming there are no errors, please turn over each card, read the number on the back, and enter it into the corresponding box on the sorting sheet. We will take a photograph or record your sorting results. We will briefly ask you a few questions, particularly regarding the cards you placed at the extreme ends (+4 and -4) and ask you to discuss the reasons behind your choices.

**Important** **Principles** **(Gentle** **Reminders)**

Forced Distribution: We understand that some cards are difficult to choose between, but for statistical purposes, you must place them according to the designated number of spaces (for example: +4 can only hold exactly 2 cards, no more and no less).

Relativity: If you feel you agree with all the cards, please place those with a “relatively weaker level of agreement” towards the left (the negative score direction). This does not mean you oppose them; it merely indicates they are relatively less important to you.

Thank you again for your cooperation and support！

**Supplementary Table S4: Post-Sort Semi-Structured Interview Guide.**

| **No.** | **Interview Question / Prompt** |
| --- | --- |
| 1 | Reflecting on the sorting process you just completed, which statements placed at the extreme ends (most agree and most disagree) best represent your core perspectives? Could you briefly explain why these particular statements are so important to you? |
| 2 | I noticed that you placed statement [XXX] in the "strongly agree/strongly disagree" position. Drawing on your specific clinical experience, could you share what gave you such strong feelings about this? |
| 3 | During the sorting process, were there any specific statements or categories of statements that you found difficult to weigh against each other, or where you felt a contradiction? How did you ultimately make your decision? |
| 4 | Did your years of clinical experience, your specific ICU subspecialty (e.g., Medical ICU, Surgical ICU), or a recent typical case influence your perspective on certain issues? For example, your earlier thoughts regarding [XXX]. |
| 5 | Do you have any practical tips or successful experiences regarding effective analgesia and sedation management that you would like to share? |
| 6 | Could you share a specific example of a particularly difficult case involving analgesia and sedation management? |
| 7 | Regarding analgesia and sedation management, or the Q-sorting process we just completed, is there anything we haven't covered that you would particularly like to add or elaborate on? |

**Supplementary Table S5. CFIR-informed characterization of the three factor viewpoints.**

| Factor viewpoint | Salient CFIR domains | Key CFIR constructs | Representative Q-statements | CFIR-informed characterization |
| --- | --- | --- | --- | --- |
| **Factor 1: Professionally Confident and Responsibility-Driven** | Characteristics of Individuals; Inner Setting | Knowledge & Beliefs; Other Personal Attributes; Structural Characteristics & Resources | S5, S10, S11, S13–S16, S47 | This viewpoint was characterized primarily by strong professional beliefs regarding analgesia and sedation management, including perceived competence, professional responsibility, and work-related fulfillment. In contrast, workload- and time-related structural constraints were comparatively less salient within this factor. This configuration supported its characterization as **Professionally Confident and Responsibility-Driven**. |
| **Factor 2: Experience-Driven with Perceived Organizational Support Gaps** | Characteristics of Individuals; Inner Setting; Process | Other Personal Attributes; Networks & Communications; Implementation Climate; Readiness for Implementation; Reflecting & Evaluating; Executing | S4, S6, S17, S25, S37, S39, S41 | This viewpoint combined a strong reliance on experiential clinical judgment with perceived limitations in physician–nurse consensus, departmental prioritization, and previous formal preparation. At the same time, participants positively valued competency assessment and situational adaptation during clinical implementation. Together, these individual-, organizational-, and process-related influences supported its characterization as **Experience-Driven with Perceived Organizational Support Gaps**. |
| **Factor 3: Systemically Isolated and Communication-Restricted** | Inner Setting; Outer Setting; Process | Networks & Communications; Structural Characteristics & Resources; Patient Needs & Resources; Reflecting & Evaluating | S18, S19, S24, S27, S37, S43 | This viewpoint emphasized perceived discontinuities in physician–nurse communication and handover information, structural constraints associated with the ICU environment, and influences related to patients’ or family members’ understanding of clinical care. Participants also placed relatively low value on regular competency assessment within this configuration. These communication- and context-related influences supported its characterization as **Systemically Isolated and Communication-Restricted**. Given that this factor was defined by only two participants, the characterization should be regarded as preliminary. |

Note: CFIR, Consolidated Framework for Implementation Research. The CFIR domains and constructs shown in this table were based on the original mapping of the 50 Q-statements presented in Supplementary Table S3. Representative statements were selected because their distinguishing and/or extreme placements contributed substantively to the interpretation of each factor; detailed Z-scores and Q-sort values are presented in Table 3. CFIR was applied as an interpretive framework after statistical factor extraction and did not determine the number or composition of the factors. Factor labels were derived from the overall Q-sort configuration together with participants’ post-sort explanations rather than directly from CFIR domain or construct names.

**Supplementary S6. Idealized Q-sort array for Factor 1**
